# Effect of High Molybdenum Diet on Copper Status, Growth Performance, Blood Metabolites, Select Liver and Kidney Minerals, and Immune Responses of Boer Crosses

**DOI:** 10.3390/ani14111604

**Published:** 2024-05-29

**Authors:** Sandra G. Solaiman, Kyla A. Beguesse, Byeng R. Min

**Affiliations:** 1 Department of Agricultural and Environmental Sciences, Tuskegee University, Tuskegee, AL 36088, USA; bmin1@tuskegee.edu; 2 Department of Biological Sciences, North Carolina State University, Raleigh, NC 27607, USA; kabegues@ncsu.edu

**Keywords:** average daily gain, goats, Cu/Mo, carcass, immune responses

## Abstract

**Simple Summary:**

Trace minerals like copper and molybdenum are essential for overall animal health and play crucial roles in metabolic reactions involving tissues and organs. A major factor affecting the absorption and utilization of trace minerals is the interactions that occur between them. A copper deficiency induced by a high molybdenum concentration in the diet (when a sheep trace mineral mix high in Mo is fed to goats) is detrimental, resulting in tremendous economic losses for the goat production industry. An experiment was conducted to determine the effect of an induced copper deficiency by increasing molybdenum on the growth performance, feed efficiency, carcass characteristics, blood metabolites, and immune responses in goats. Eighteen Boer crosses goat kids housed individually were randomly assigned to three treatment groups; (1) control, (2) 5 ppm Mo, and (3) 10 ppm Mo, with 0, 5, and 10 mg/kg of molybdenum added to the grain mix, respectively. The results indicate that low-copper diets with increasing molybdenum did not negatively affect the feed efficiency, animal performance, blood metabolites, or carcass characteristics; however, it did alter the liver’s copper and iron concentration as well as immune responses. These results suggest that additional Mo reduced the copper and iron in the liver for the duration of this experiment, and negatively impacted the immune response.

**Abstract:**

This study examined the effects of elevated molybdenum (Mo) in goat diets on the growth, blood parameters, and immune responses in goats. Eighteen Boer crosses goats (BW = 25.6 ± 1.03 kg) were randomly assigned to three treatment groups: (1) control (no additional Mo), (2) 5 ppm Mo, and (3) 10 ppm Mo as ammonium molybdate was added to the grain mix. Animals were fed a 50:50 hay:grain diet ad libitum twice daily. Daily feed refusals were monitored, and intake was adjusted weekly. Body weights were recorded every 14 days and blood samples were collected on the second week of every month to determine Cu, Mo, Fe, Zn, and other blood metabolites. After 85 days, animals were humanely euthanized and carcass traits were measured. Liver, longissimus muscle area, and kidney samples were collected postmortem. Liver Cu (*p* < 0.003), blood triacylglycerides (*p* < 0.03), and serum total protein (*p* < 0.03) levels were reduced; the liver (*p* = 0.07) and kidney (*p* < 0.001) Mo concentrations were increased; and the immune response was decreased linearly (*p* < 0.01) with additional Mo. Low levels of Cu with increasing Mo levels in the diet did not negatively impact animal performance or blood metabolites, in the duration of this study (85 days); however, it lowered the liver Cu, Fe, and immune responses in goats.

## 1. Introduction

Goat producers feeding sheep mineral mixes high in Mo to goats claim that it causes Cu deficiency in goats, reduces the immune response, and increases animals’ parasite load [1]. An interaction between copper (Cu), molybdenum (Mo), and sulfur (S) has been recognized [2]. A high concentration of Mo has been reported to interfere with the absorption of Cu, which can potentially lead to Cu deficiency [3]. Thiomolybdates (mono-, di-, tri-, and tetrathiomolybdates) are known to be potent antagonists of the Cu metabolism and can cause a Cu deficiency in ruminants [4]. Thiomolybdates are formed by a molybdate interaction with sulfide which is produced by rumen microorganisms through sulfate and also the degradation of sulfur-containing amino acids [5]. Ward et al. [6] demonstrated a reduction in plasma Cu by Mo and S supplementation in a study performed on crossbred steers for 98 days. In a study conducted on Angus bull calves, Mo supplementation decreased liver Cu concentrations [7]. Also, a Cu deficiency in yaks resulted from increased levels of naturally occurring Mo in the soil and forages in China [8]. Biochemical influences on the Cu metabolism that were associated with thiomolybdates include (1) the enlarged biliary secretion of Cu from liver stores; (2) the effective requisite of Cu to plasma albumin, which results in a reduced biochemical process; and (3) the removal of Cu from metalloenzymes [9]. It has been reported that adding 3.0 g of S and 4.0 mg of Mo/kg of the diet to the basal diet that contains 1.0 g of S and 0.5 mg of Mo/kg of the diet decreased the Cu metabolism by 40 to 70% [10], while a moderately high level of S (2.7 g S/kg of the diet) in the diet, with increasing dietary Mo from 5 to 10 mg of Mo/kg of the diet, did not further reduce the Cu status in steers [11], which suggests that the synthesis of thiomolybdates may plateau with relatively moderate levels of Mo in the diet. The impact of a copper-deficient diet with elevated Mo and its effects on performance and immune responses in goats are poorly understood. To validate this hypothesis, an experimental study was conducted to determine the impact of increasing levels of Mo with low levels of Cu in diets on the growth performance, blood metabolites, carcass characteristics, and immune responses in meat goats.

## 2. Materials and Methods

This experiment complied with the Tuskegee University Institutional Animal Care and Use Committee regulations (Protocol # R066-7-1).

### 2.1. Preparation of Experimental Animals

Boer × Spanish goat kids were purchased from a local market in Alabama. Animals were treated prophylactically for shipping fever with Oxybiotic IM, internal parasites with Cydectin, an Avermectin class of anthelmintics for cattle (Moxidectin; Fort Dodge Animal Health, Fort Dodge, IA, USA) by oral drench at a dosage rate of 1 mL per 10 kg BW. Animals were vaccinated subcutaneously with 2 mL of Clostridium Perfringens type C- and D-Tetani Bacterin Toxoid (Bayer: Bayer Corp., Animal Health, Shawnee Mission, KS, USA). Animals were quarantined for 26 days. After periodic examination of each animal throughout the quarantine period by the attending veterinarian, Tetradure 300 (oxytetracycline; Merial Ltd., Duluth, GA, USA), given at a dosage of 0.75 mL/11.4 kg intramuscularly for treatment of shipping fever and Valbazen, and a broad-spectrum anthelmintic dewormer (Albendazole; Pfizer Animal Health, New York, NY, USA) given orally at a dosage of 1.3 mL/11.4 kg for treatment of internal parasites, were administered as needed. Animals’ health and wellbeing was monitored throughout the study.

### 2.2. Experimental Diets

In this experiment, animals were given 50% formulated grain mixture (grain mix) and 50% Bermudagrass hay (BrGH) diets (Table 1). Diets were gradually adjusted to a 50:50 grain-to-hay ratio over 10 days from September 6 through 15. On September 16, animals were placed on permanent diets. The grain mix for control treatment was prepared and mixed at a local feed mill with added trace mineral mix. To induce copper deficiency and represent the feeding practices of local producers, treatment diets were prepared with grain mix, but without trace mineral mix, and with added 5 ppm and 10 ppm molybdenum as Ammonium Molybdate ((NH_4_)_6_Mo_7_O_2. 4_(H_2_O)). The ingredient composition of the grain mix is presented in Table 1.

Bermudagrass hay was chopped to approximately 2 to 3 inches in length using a hay chopper and stored in a covered wooden box. According to NRC 2007 [12], feed intake was calculated to meet and exceed nutrient requirements for maintenance, plus 100 to 150 g gain per day and a 5 to 10% refusal. Grain mix and BrGH were fed separately (according to the predefined ratio of 50:50), and fresh feeds were provided twice a day at 0600 h and 1530 h ad libitum (unrestricted). Daily feed intakes were recorded by measuring feed offered minus feed refused. Refusals (grain mix plus BrGH) were collected daily prior to the afternoon feeding, and feed intake was adjusted weekly. If refusal was more than 10%, feed offered was decreased. When refusal was less than 5%, the feed offered was increased. When feed offered was adjusted according to the amount of refusals in the previous week, the forage-to-concentrate ratio was always maintained at 50:50. Intake was calculated based on actual consumption of hay and grain, which may have deviated from the 50:50 grain-to-hay ratio offered, due to BrGH being refused before the grain. Ad libitum access to water was provided throughout the study.

### 2.3. Experimental Design and Data Collection

Eighteen Boer crossed goat kids with average body weight (BW) of 25.6 ± 1.03 kg and average age of 4 to 5 months old were stratified by body weight and randomly assigned to three treatment groups (n = 6). Treatments consisted of (1) control (no additional Mo), (2) 5 ppm Mo, and (3) 10 ppm Mo as ammonium molybdate tetrahydrate added to the grain mix. Animals were housed indoors in individual pens measuring 1.8 × 2.1 m^2^. The pens had raised mesh floor type, which allowed feces and urine to be flushed daily into a lagoon system. Baseline blood samples were obtained via jugular venipuncture before initiation of the study (day 0) and analyzed using Cell-Dyn 3700 OS open system (Abbott Diagnostics, Chicago, IL, USA) for complete blood counts, and ACE Chemical Analyzer (Alfa-Wassermann, West Caldwell, NJ, USA) for serum chemistry parameters at the Tuskegee University Clinical Diagnostic Laboratory.

Daily feed intake and refusals of grain mixes and hay were monitored separately and recorded, and intake was adjusted weekly for 85 days. General health parameters obtained every four weeks included vital body signs of respiration rate (RR), heart rate (HR), ruminal contractions (RC), body condition score (BCS; scale of 1 to 5), and rectal temperature (RT). Body weights were obtained every two weeks and recorded after 4 h of feed and water restriction. Blood samples were collected from each animal on the second week of every month to determine trace minerals (Cu, Mo, Fe, and Zn) and other blood metabolites. Two mL EDTA vacutainer blood collection tubes were used for the complete blood count. Ten mL non-EDTA vacutainer blood collection tubes were used for serum blood metabolites and lipid profile. Six mL acid-washed, non-EDTA vacutainer blood collection tubes were used for serum Cu, Fe, Zn, and Mo analysis. Blood samples were placed on ice immediately after collection and transported to Tuskegee University Clinical Diagnostics Laboratory for further preparation.

The experiment consisted of a 10-day adjustment and an 85-day experimental phase. Individual goat average daily gain (ADG) was calculated as the difference between initial and final BW over the interval of the performance phase. Average dry matter intake (DMI) was calculated on a dry matter (DM) basis over the performance period for each goat and consisted of grain and BrGH DM intake. The gain efficiency ratio was determined by dividing ADG over DMI. Composite samples of formulated grain mix and BrGH were collected weekly, labeled according to the month collected, placed in secure zip-lock bags, and stored in a dry area at room temperature until further chemical and mineral analysis.

Animals were transported to Auburn University Lambert-Powell Meat Lab after 85 days and harvested according to USDA-approved methods. After harvest, hot carcass weight (kg) (HCW) was determined. Carcasses were rinsed and chilled for 24 h to an internal temperature of 4 °C. The following quality parameters were determined by a certified USDA grader 24 h after harvest: back fat (BF; fat depth over the longissimus muscle between the 12th and 13th ribs, cm); dressing percentage (DP; HCW/live wt.); chilled carcass weight (CCW; kg); carcass shrink (difference in CCW and HCW; kg); adjusted body wall fat (BWF; cm); adjusted fat thickness (ADFT; cm); and longissimus muscle area (LMA; cm^2^; measured using a rib eye grid). The Institutional Meat Purchase Specifications for Fresh Goat, Series 11 (IMPS; USDA, 2001) [13], was used to report carcass selection criteria 24 h after harvest. Liver (right lobe) and kidney samples were obtained from each animal postmortem and immediately placed in plastic whirl-pack bags to be stored on ice for mineral analysis. After carcass grading and measurement, a longissimus muscle sample, 1.5 cm in depth (approximate weight 50 to 100 g), was sliced from the right side of the carcass dorsal from the area of BF measurement. Samples were placed in vacuum-sealed bags and immediately chilled on ice. Upon arrival at Tuskegee University Nutrition Laboratory, all samples were frozen at −20 °C until further analysis.

### 2.4. Handling and Processing of Samples

Samples of each feed (grain mix and BrGH) were collected weekly and pooled to form composite samples representing each month of the performance period. Feed samples were partially dried and ground in a Thomas Model 4 Wiley mill (Thomas Scientific, Swedesboro, NJ, USA) to pass through a 1 mm mesh screen, labeled, and placed in sealed plastic containers. Ground samples were packaged and shipped to Holmes Laboratory, Inc. (Millersburg, OH, USA) for chemical analysis. A small portion (about 2 to 4 g) of liver and kidney samples were thawed, diced into small pieces, and transported (on ice) along with serum samples from each animal to Auburn University State Diagnostic Laboratory to be analyzed for Cu, Mo, iron (Fe), and zinc (Zn).

### 2.5. Laboratory Analysis

Hematology profiles (complete blood cell count) on 72 EDTA blood samples collected during this study were performed on the CELL-DYN 3700 System (Abbott Diagnostics, Chicago, IL, USA), a multi-parameter automated hematology analyzer designed to evaluate complete counts and characterization of blood cells that is widely used in clinical diagnostic laboratories.

Blood serum metabolites (blood chemistry) measurement on 72 non-EDTA samples collected were performed on the ACE Clinical Chemistry System (Alfa Wasserman, West Caldwell, NJ, USA). This is an automated benchtop clinical chemistry analyzer that is standardly used in clinical diagnostic laboratories designed to conduct comprehensive metabolic panels and internal organ functions. Trace mineral concentrations in serum samples (diluted 1:4 in deionized H_2_O) were determined by flame atomic absorption spectrophotometry (GBC 908AA, Perkin-Elmer, Wellesley, MA, USA).

Dry matter (DM), crude protein (CP), ether extract (EE), and ash (ASH) from composite samples of grain mix and BrGH were determined using standard AOAC procedures [14]. Neutral detergent fiber (NDF), acid detergent fiber (ADF), and sulfuric lignin (ADL) were determined according to Van Soest et al. [15] and modified according to Komarek [16] for use in an Ankom fiber apparatus (Ankom Technology Corp., Fairport, NY, USA).

Micromineral and trace mineral concentrations were determined using dry ashing methods, as described by Hue and Evans [17]. The concentration of Mo in the feed was determined by ashing in a muffle furnace and digested using a microwave digestion system (Model MDS 2100, CEM, Matthews, NC, USA), according to procedure outlined by Gengelbach et al. [18], and analyzed by flameless (graphite furnace) atomic absorption spectrophotometry (Perkin–Elmer model 5100; equipped a Zeeman 5100; Wellesley, MA, USA).

Samples of liver and kidney were dried at 100 °C for 48 h, weighed, and 5 g DM of each sample were ashed in a muffle furnace (SyBron Thermolyne Furnatrol I, model 18200; Dubuque, IA, USA) at 600 °C overnight. Ashed samples were digested in 10 mL of HNO_3_ over a hot plate until ash was dissolved and transferred to a plastic centrifuge tube [14]. Kidney samples were diluted to 50 mL and liver samples ranged from 50 to 150 mL dilutions with distilled water. Flame atomic absorption spectrophotometry (GBC 908AA; Perkin-Elmer, Wellesley, MA, USA) determined Cu, Fe, and Zn concentrations and reported on a DM basis.

### 2.6. Immunological Procedure

#### 2.6.1. Cell-Mediated Immunity

On day 82, an area in the lumbar region behind the neck was shaved in a 2 × 2 cm square and cleaned. Then on day 83, two skin-fold injection sites labeled A and B, were measured and then injected intradermally with 200 µL of phytohemagglutinin A (PHA). Measurement of skin folds were taken pre-injection and 24 and 48 h post-injection using scientific calipers. The PHA (0.025 g; Sigma Chemical Co., St. Louis, MO, USA) was mixed in 5 mL of phosphate buffer solution (PBS) to make a 250 μg/100 μL solution. Skin folds were measured using scientific calipers in millimeters on days 84 and 85, respectively, 24 and 48 h post-injection.

#### 2.6.2. Humoral Immunity

Humoral immunity was measured via antibody titers to chicken ovalbumin. Goats were injected intramuscularly with 2 mg of chicken ovalbumin (ova; Sigma Chemical Co., St. Louis, MO, USA) in 0.4 mL of 1:1 PBS: Freund’s incomplete adjuvant (Sigma Chemical Co., St. Louis, MO, USA) on day 64. Goats were bled via jugular venipuncture on days 78 and 85. Enzyme-linked immunosorbent assay (ELISA) was used to analyze the serum samples for IgG antibody titers to chicken ovalbumin. The 96-well plates were coated with ovalbumin (100 µL/well) in carbonate buffer (pH 9.8) and incubated overnight at 4 °C. Antigen concentration was 10 µg/mL. The following day they were placed into an incubator at 37 °C for one hour. The plates were washed three times with ELISA PBS and 0.1% Tween. To block uncoated spaces, 200 µL of 2% bovine serum albumin (Sigma Chemical Co., St. Louis, MO, USA) in ELISA PBS was added to the plates and placed into the incubator for 30 min at 37 °C. Next, the plates were washed three times with ELISA PBS and 0.1% Tween. Plates were then injected with serial dilution of goat serum in ELISA PBS and 0.1% Tween (3 wells/dilution and 100 µL/well) and incubated at 37 °C for one hour. Plates were then washed again with ELISA PBS and 0.1% Tween. The wells were individually injected with 100 µL of Rabbit Anti-goat peroxidase conjugate (Sigma Chemical Co., St. Louis, MO, USA) 1:3000 dilution in ELISA PBS and Tween (Sigma Chemical Co., St. Louis, MO, USA). After incubation at 37 °C for one hour, plates were washed with ELISA PBS and Tween. As a color substrate, 2,2′-Azino-bis (3-ethylbenzothiazoline-6-sulfonic acid) was added to each well and allowed to develop for 30 min. After developing, plates were read under an ELISA microplate reader with a filter set at 405 nm at a serum dilution of 1:256. Results are expressed as a titer, a measurement of the amount of a substance (usually an antibody concentration) in a solution that correlates to the highest dilution factor required to yield a positive ELISA reading.

### 2.7. Statistical Analysis

Data collected in this experiment were analyzed by the Proc Mixed procedure of SAS Version 8 [19]. Performance data collected were averaged over the 12-week collection period and analyzed as a complete randomized design with increasing Mo levels as a source of variation. The Mo level effects were tested by a polynomial regression using orthogonal contrast for equally spaced treatments [20] estimated by the Proc Mixed procedure of SAS. The least-square means procedure was used when appropriate to compare differences between treatment means. Differences were declared significant at *p* < 0.05, unless otherwise indicated.

## 3. Results and Discussion

### 3.1. Chemical Composition of Experimental Diets

The chemical composition of the experimental diets is listed in Table 2. The basal diet was a cracked corn, alfalfa pellets, whole oats, and soybean meal-based diet with a concentrate-to-BrGH ratio of 50%:50% as the feed basis. The grain mix for the control group contained CP and total digestible nutrients (TDN) contents of 20.2 and 80.8%, respectively. The other nutrient contents in the experimental diets, including NDF, ADF, ADL, fat, non-fibrous carbohydrates (NFC), and starch, were similar among treatments. The nutrient contents of BrGH were similar to the reported values by the NRC [12]. The grain mix for the control group contained an Fe content of 328 ppm, Zn of 134 ppm, and Mn of 118 ppm. All the treatment diets met the requirements of Fe, Zn, and Cu for growing meat goats according to the NRC [12], except for the fact that diets with higher Mo contents of 5 and 10 ppm had lower ratios of Cu to Mo. The actual Mo contents of grain mixes when analyzed were 2.52 ppm for the control, and 6.82 ppm and 16.5 ppm for 5 and 10 ppm Mo grain mixes, respectively, which slightly deviated from intended levels of 5 and 10 ppm grain mixes. In the present study, the Cu-to-Mo ratio was 10.9 to 1, 0.88 to 1, and 0.42 to 1 for the control, 5, and 10 ppm Mo grain mixes, respectively, to ensure Cu deficiency. It is essential to determine the total dietary concentration of Mo, S, and Cu so that a better elucidation of Cu bioavailability can be made. In the present study, we did not measure the S content in the diet. However, the level of S in the rumen depends on the dietary CP level in the ration and the protein solubility [21]. In the present study, the dietary CP levels and sources were similar among the treatments, thus we assumed the diets had similar S values. The control diet, having added the trace mineral mix, had a higher Cu-to-Mo ratio, whereas diets containing 5 and 10 ppm of added Mo had low ratios.

### 3.2. Feed Intake and Growth Performance

The mean body weight (BW), average daily gain (ADG), cumulative DMI, DMI as %BW, and the gain efficiency (gain:feed intake ratio, G:F) in goats fed increasing levels of Mo throughout the performance period (85 days) are presented in Table 3. There was no difference in the initial BW and final BW between the treatment groups (*p* > 0.05). The ADG over the performance period (85 days) ranged from 125 g/day for the control group, 130 g/day for the 5 ppm Mo group, and 143 g/day for the 10 ppm Mo group. However, in the present study, most probably due to variation in the means and small sample size, there was no treatment effect for any of the parameters analyzed for growth performance including G:F.

There was no significant difference in the total DMI, DMI as % BW, and hay intake among treatment groups, but the grain intake linearly increased (*p* < 0.01), probably largely due to the increase in the 10 ppm Mo diet. However, considering SEM, and the similarity of the 5 ppm and 10 ppm diet composition (the only difference was the additional Mo in 5 vs. 10 ppm), the increase in the grain mix intake may be statistical as well as biological. Frank et al. [3] reported that the feed intake for the Cu-deficient groups appeared to decrease, while that of the Cu-adequate control and chromium (Cr)-deficient groups continued to increase DMI. The ultimate increase in the grain intake for Cu-deficient groups in this study may be caused by supplementation with Mo in the form of ammonium molybdate [3].

### 3.3. Animal Health Parameters

The effects of added dietary Mo on the animal health parameters of goat kids are presented in Table 4. The consequence of an unnecessary Mo intake and Cu deficiency and an imbalance of these two metal ions and S in the feed or digested from S-containing amino acids in the rumen is a complex nutritional problem in ruminants [22,23]. However, in the present study, there was no difference in animal health parameters among treatments for the heart rate, respiration rate, ruminal contraction (per min), body temperature, and body condition score (on the scale of 1 to 5) in meat goat kids. When compared to sheep and cattle [24], goats have less clinical lesions with the Cu or Cr deficiencies [25] and are more resistant when they have been subjected to a rather severe trace element deficiency and/or imbalance [26].

### 3.4. Blood and Liver Metabolites and Hemogram

The mean concentrations for blood metabolites and the hemogram are listed in Table 5. Among the dietary treatment groups, there was no difference in the blood serum enzymes (ALT, AMYL, and CK), albumin, cholesterol, BUN, glucose, and the hemogram. However, TRIG, total protein, and red blood cells, although within the normal range, decreased linearly (linear; *p* = 0.05) as levels of Mo increased in the diet. These results contrast with the findings of Al-Othman et al. [27] where increasing levels of Mo in rats disrupted the role that Cu plays in the lipid metabolism resulting in increasing the concentration of serum TRIG. However, there was no significant difference in cholesterol among the treatment groups in our study, contrasting with Xiao-Yun et al. [8] where elevated serum cholesterol was reported in yaks in the North of the Qing Hai–Tibetan Plateau of China with a severe Cu (level) deficiency caused by a high level of naturally occurring Mo in the soil and forages. In their study, Cu/Mo ratios of 1.34 ± 0.36 vs. 8.12 ± 1.31 μg/g for affected and unaffected forage areas were reported, respectively. The mean concentrations of Cu in the blood and hair from the affected yaks were 0.29 ± 0.17 and 3.51 ± 1.12 μg/g, respectively, compared with 0.85 ± 0.24 and 6.42 ± 1.21 μg/g for the controls. Additionally, yaks developed hypochromic microcytic anemia and had a low ceruloplasmin level in their blood [8]. In the present study, the ratios of Cu to Mo were 10.9 to 1 for the control, 0.88 to 1 for 5 ppm Mo, and 0.42 to 1 for 10 ppm Mo diets for the 85-day experimental phase. The goats did not exhibit any signs of Cu deficiency during the experiment.

According to Solaiman et al. [28], supplemental Cu did not affect serum triglycerides; however, NEFA decreased as the supplemental Cu increased. The findings of Engle et al. [29] and Lee et al. [30] also showed that triglycerides were unaffected by Cu supplementation in steers; however, in the findings of Bakalli et al. [31], plasma triglycerides were decreased in broilers fed 250 mg of Cu/kg of DM. As mentioned above, [28,29,30,31] reports on the effects of Cu supplementation (or lack of) on cholesterol, triglycerides, and NEFA are inconsistent. The animal species, breed, plasma vs. serum used, individual animal variability, number of animals tested, diets fed, level of supplemental Cu, or concentration of other minerals such as Zn, Mo, or S in the diet may be contributing factors [28].

### 3.5. Blood, Liver, and Kidney Minerals

Ninety percent of plasma Cu is bound to globulin, ceruloplasmin, and albumin, acting as a true transporter of Cu, and a small portion is bound to amino acids [32]. The mean concentrations for Cu, Zn, Fe, and Mo in the serum, liver, and kidneys are listed in Table 6. In the present study, the serum albumin and Cu concentration were not affected by treatments, indicating that 5 to 10 ppm Mo supplementation in the grain mixture did not negatively affect serum Cu metabolites during the 85-day experimental phase of the study. However, serum Fe tended to decrease (*p* = 0.09), which may have resulted in lowered RBCs (*p* = 0.02). A normal Cu concentration is suggested to be 0.7 to 1.5 mg/L [32], similar to the current study ranging from 1.05 to 1.08 mg/L. A plasma/serum Cu concentration consistently below 0.5 mg/L was strongly correlated with a liver Cu concentration less than 40 ppm on a DM basis [33]. In our study (Table 6), the Cu concentration in the liver was linearly decreased (*p* = 0.003) with increasing Mo supplementation, but the Cu (120 to 380 ppm) concentration in the liver was within normal ranges [34,35]. To date, the normal reference range for Cu in the goat liver that is widely accepted in veterinary medicine for the diagnosis of Cu deficiency is 25 to 150 mg/L on a wet weight basis [35,36]. According to the NRC [12], a liver Cu concentration of 20 mg/kg DM is considered the suggested deficient value. Although the liver Cu concentration in the present study decreased rapidly, after 85 days, the highest Mo treatment group still had a liver Cu concentration well above 20 mg/kg DM, suggesting no deficiency values were observed after 85 days of feeding high levels of Mo in the grain mixture. The current study is the first study to evaluate the concentration of Mo in the liver and kidney of goats; therefore, no comparisons can be made at this time. Liver Cu and Fe decreased (*p* < 0.001), and Mo tended to increase (*p* = 0.07) with no changes in the Zn concentration as the Mo intake increased in the diet. Kidney Mo in goat kids fed the treatment diets increased (*p* = 0.001), with numerically higher concurrent increases in Cu and Zn. This perhaps could be attributable to the considerable variation and smaller sample size. The Fe level did not differ in the kidneys when Mo increased in the diet. Although blood metabolites and liver and kidney mineral changes were observed in Mo-supplemented groups compared with the control group, no conclusive changes were observed from a physiological and nutritional point of view in this study. Similarly, although the mineral concentrations were significantly altered in the kidneys of Cu- and Cr-deficient goat kids with Mo supplementation, no marked changes were observed in the kidneys of meat goats [3].

No hepatic alterations were detected in our study, supported by clinical chemistry findings with normal ALT, AMYL, CK, and hemogram levels.

### 3.6. Immune Responses

The effects of elevated Mo on humoral and cell-mediated immune responses are presented in Table 7. Goats receiving elevated Mo in the grain mixture exhibited linearly lower humoral (ovalbumin, *p* = 0.001) and cell-mediated (PHA, *p* = 0.002) immune responses. Mo may have an associative interaction to interfere with the absorption of other minerals such as Cu and Fe, which can potentially lead to a copper deficiency [4,6,7] and lower immunity. Elevated dietary Mo, with limited Cu in the diet, negatively influenced the immune function of growing goat kids. The decreases in the humoral and cell-mediated immune responses suggests that growing goat kids consuming the treatment diets (with Cu:Mo ratios of 0.88:1 and 0.42:1) may be more susceptible to disease than goats consuming a diet with a higher concentration of Cu, as in the control diet (Cu:Mo ratio of 10.9:1). It has also been reported that the liver lesions in goats in the Cu- and Cr-deficient groups were more clearly developed, probably due to a Cu deficiency and molybdenosis [25]. Solaiman et al. [26] reported that goats are less sensitive to high Cu supplementation than sheep, suggesting that goats may have more tolerance to higher levels of Cu than sheep or cattle [25,37]. This may indicate that goats may have higher Cu requirements per se than sheep and cattle. The quantity of Cu needed in the goat’s diet is presently set at 15–25 ppm, depending on the physiological state of the animal [12]. Consequently, many commercially produced goat feeds are formulated to contain 25 to 30 ppm Cu/kg DM [37]. In the present study, goats received 27.5 ppm supplementation in the control diet, sufficient for the daily Cu requirement in meat goats. Mo treatment groups had much lower levels of Cu in their diets (6.0 to 7.0 ppm Cu/kg DM), suggesting a possibility that having a high concentration of Cu in the liver may have helped to maintain the serum Cu concentration within the normal range and prevent the full development of a Cu deficiency within the experimental period of 85 days.

### 3.7. Carcass Characteristics

The carcass measurements and selection criteria obtained in this study are shown in Table 8. No significant differences were observed in the HCW, CCW, shrink, DP, ADFT, LMA, BF, and selection criteria. The measurements for the HCW were 17.0 kg for both the control and 5 ppm Mo group and 17.5 kg for the 10 ppm Mo group. The cold carcass weights were 16.5 kg for the control group, 16.4 kg for the 5 ppm Mo group, and 16.9 kg for the 10 ppm Mo group. Most of the current studies involving ruminants have established the purpose of their research as evaluating the positive effects of increasing levels of Cu on carcass characteristics. The published literature illustrating how Cu and Mo interactions affect carcass characteristics in goats is minimal. Koo et al. [37] observed increased cholesterol synthesis in Cu-deficient rats fed Mo supplementation at 5 mg Mo/kg DM and tended (*p* = 0.11) to increase in BF. One could speculate that the contrast between Koo et al. [38] and the results observed in this study is due to the fact that the animals in this study had a higher Cu status initially, preventing the depletion of liver Cu concentrations that would lead to a deficiency. Back fat is one of the three major factors affecting the DP [39]. In a study performed by Engle et al. [29] on steers fed Cu/kg DM diets at 0 mg (no supplemental Cu), 20 mg (from Cu sulfate, Cu citrate, Cu proteinate, and tribasic Cu chloride), and 40 mg (from Cu sulfate) for 101 and 121 days, lowered (*p* < 0.05) HCW and BF were observed, but the DP and LMA were unaffected by the dietary Cu treatments. The BWF observed in this study tended to be higher (quadratic; *p* = 0.06) in the 5 ppm Mo group (0.74 ± 0.09 cm) when compared to the control (0.51 ± 0.09 cm) and 10 ppm Mo group (0.53 ± 0.09 cm). The results observed in the control group (no added Mo) for this study agreed with the findings of Solaiman et al. [28,40], demonstrating Boer × Spanish wether kids supplemented with 100 mg Cu/day and 200 mg Cu/day for 112 days tended to decrease in their BWF (linear; *p* = 0.08).

## 4. Conclusions

The results from this study indicate that diets with elevated levels of Mo without increasing Cu did not negatively affect DMI, feed efficiency, animal performance, blood metabolites, and carcass characteristics in 85 days; however, they did alter the immune responses in goat kids. The liver copper and iron concentrations decreased significantly compared to the control group, with no change in zinc; therefore, with a longer duration of the experimental period, the animals may have exhibited a copper deficiency. Kidney molybdenum was increased, indicating the kidney as a possible route of Mo excretion.

This experiment clearly demonstrates that although goats can perform well and look healthy, with proper vital signs, serum metabolites, and carcass characteristics, they can still be immune-compromised and show signs of deficiencies later. There is no reason that humoral and cell-mediated immune responses cannot be used to refine the mineral requirements in goats, in addition to improving their productive performance. Maybe it is advisable to use the immunity in goats as a measure of their productivity or nutrient sufficiency/deficiency.

## Figures and Tables

**Table 1:** Ingredient composition (as fed basis) of grain mixes fed to goats.

Item	Grain Mix (ppm)		
	0 ppm Mo	5 ppm Mo	10 ppm Mo
Ingredients, % as is			
Cracked corn	44.0	44.0	44.0
Alfalfa pellets	15.5	15.5	15.5
Whole oats	17.0	17.0	17.0
Soybean meal, 49%	17.5	17.5	17.5
Molasses	5.0	5.0	5.0
Dicalcium phosphate	0.5	0.5	0.5
Trace mineral mix	0.5	0.00	0.00
Ammonium molybdate (54.1% Mo), mg	0.0	935	1870

**Table 2:** Chemical composition (% DM) of experimental diets (grain mix and hay) fed to goats.

Item	Grain Mix (Mo, ppm)				
	0	5	10	Hay ^1^	SEM
Nutrient contents, % DM					
DM	87.2	87.2	87.8	89.7	0.21
CP	20.2	19.0	21.6	10.2	0.88
NDF	18.6	19.9	18.4	69.5	0.97
ADF	11.5	12.4	11.3	34.0	0.60
ADL	0.94	1.42	1.05	2.85	0.23
Ether extract	3.57	3.67	3.68	1.25	0.11
NFC	51.7	52.3	50.5	15.8	0.80
Starch	49.2	49.7	48.0	2.37	0.57
TDN	80.8	80.4	80.7	59.7	0.56
Ash	6.14	5.41	6.20	6.56	0.33
Trace minerals (ppm)					
Fe	328	242	259	151	24.9
Zn	134	61.5	73.0	22.7	7.0
Mn	118	67.5	71.0	68.2	6.61
Cu	27.5	06.0	7.0	7.25	1.67
Mo Calculated Cu:Mo Ratio	2.52 10.9:1	6.82 0.88:1	16.5 0.42:1	1.72	0.74

^1^ Hay = Bermudagrass hay. DM = dry matter; CP = crude protein; NDF = neutral detergent fiber; ADF = acid detergent fiber; ADL = acid detergent lignin; NFC = non-fiber carbohydrate; NFC was calculated by difference [100 − (%NDF + % CP + % fat + % ash)]; TDN = total digestible nutrients; Fe = Iron; Zn = Zinc; Mn = Manganese; Cu = Copper; Mo = Molybdenum; SEM = standard error of the mean.

**Table 3:** Effects of added dietary molybdenum on dry matter intake (DMI), gain:feed intake (G:F) ratio, and growth performance of goat kids for 85 days.

Item		Mo, ppm		SEM	Linear ^a^	Quad
	0	5	10			
Animal Performance						
Initial BW, kg	26.0	25.9	25.1	1.04	0.57	0.81
Final BW, kg	36.6	37.0	37.3	1.25	0.69	0.97
ADG, g	125	130	143	14.7	0.43	0.88
DMI, kg	1.07	1.00	1.02	0.01	0.87	0.82
Hay, g	481	479	481	4.10	0.96	0.88
Grain, g	525	523	542	3.19	0.01	0.21
DM, % BW	3.58	3.69	3.64	0.17	0.89	0.67
Grain intake, % DM	53.1	53.1	53.7	0.42	0.32	0.57
G:F ratio	0.13	0.13	0.14	0.01	0.36	0.98

^a^ Based on orthogonal contrast for equally spaced treatments. BW = body weight; ADG = average daily gain; DMI = dry matter intake; G:F = gain:feed intake ratio.

**Table 4:** Effect of added dietary molybdenum on health parameters of goat kids.

Item		Mo, ppm		SEM	Linear ^a^	Quad
	0	5	10			
Heart rate	106	105	97.4	4.59	0.19	0.52
Respiration rate	23.8	23.1	23.1	0.94	0.62	0.77
Ruminal contractions/min	2.52	2.14	2.16	0.19	0.76	0.08
Body temperature, °C	38.7	38.7	38.9	0.08	0.12	0.36
Body condition score (1 to 5)	3.82	3.89	4.12	0.15	0.16	0.66

^a^ Based on orthogonal contrast for equally spaced treatments.

**Table 5:** Effects of added dietary molybdenum on blood metabolites and hemogram of goat kids.

Item		Mo, ppm		SEM	Linear ^a^	Quad
	0	5	10			
Blood serum metabolites						
ALT, IU/L	15.6	15.8	14.0	1.01	0.26	0.45
AMYL, IU/L	52.7	35.6	42.9	7.61	0.37	0.19
CK, IU/L	185	246	202	25.3	0.63	0.10
TRIG, mg/dL	40.5	29.0	29.5	3.44	0.03	0.16
Cholesterol, mg/dL	64.3	60.3	61.1	2.43	0.36	0.41
BUN, mg/dL	19.8	18.1	19.3	0.78	0.65	0.12
Glucose, g/dL	61.5	58.5	62.0	1.07	0.72	0.01
Total protein, g/dL	6.46	6.13	6.18	0.09	0.03	0.09
ALB, g/dL	2.47	2.33	2.46	0.05	0.14	0.25
Hemogram						
RBC, 10^6^/µL	13.9	13.4	13.5	0.14	0.02	0.12
Hemoglobin, g/dL	10.2	9.71	9.96	0.17	0.29	0.07
Hematocrit, %	26.3	26.3	25.5	0.35	0.11	0.37
MCV, fL	18.9	19.6	18.9	0.26	0.99	0.04
MCH, pg	7.33	7.23	7.40	0.08	0.56	0.17
MCHC, %	38.9	37.0	39.3	0.67	0.68	0.02
WBC, 10^3^/µL	11.9	13.9	12.0	0.62	0.87	0.02
As a % of WBC						
Neutrophils	50.9	48.4	47.3	1.96	0.19	0.77
Lymphocyte	39.6	44.4	44.2	1.93	0.10	0.30

^a^ Based on orthogonal contrast for equally spaced treatments. ALT = alanine aminotransferase; AMYL = amylase; CK = creatinine kinase; TRIG = triglycerides; BUN = blood urea nitrogen; ALB = albumin; RBC = red blood cell; MCV = mean corpuscular volume; MCH = mean corpuscular hemoglobin; MCHC = mean corpuscular hemoglobin %; WBC = white blood cells.

**Table 6:** Effects of added dietary molybdenum on select tissue mineral concentrations of goat kids.

Item		Mo, ppm		SEM	Linear ^a^	Quad
	0	5	10			
Serum minerals						
Ca, mg/dL	9.40	9.24	9.32	0.09	0.54	0.32
Na, mEq/L	143	142	142	0.42	0.05	0.30
K, mEq/L	5.0	4.84	4.81	0.09	0.15	0.60
Cl, mEq/L	111	111	111	0.48	0.67	0.97
Cu, mg/L	1.08	1.05	1.06	0.05	0.78	0.70
Fe, mg/L	1.40	1.33	1.17	0.10	0.09	0.84
Zn, mg/L	0.59	0.60	0.65	0.02	0.09	0.54
Liver, ppm						
Cu	380	152	120	52.4	0.003	0.15
Mo	5.17	4.93	6.03	0.32	0.07	0.11
Fe	190	137	132	10.9	0.002	0.09
Zn	94.7	88.3	102	7.24	0.50	0.28
Kidney, ppm						
Cu	12.6	13.2	149	75.1	0.22	0.47
Mo	2.38	3.02	7.38	0.88	0.001	0.10
Fe	142	127	148	9.72	0.63	0.14
Zn	63.0	68.7	157	46.6	0.17	0.48

^a^ Based on orthogonal contrast for equally spaced treatments.

**Table 7:** Effect of added dietary molybdenum (Mo) on humoral and cell-mediated immune response in goat kids.

Item		Mo, ppm		SEM	Linear ^a^	Quad
	0	5	10			
Ovalbumin	86.7	46.7	33.5	11.5	0.001	0.35
PHA, mm						
Day 83	3.25	3.36	3.38	0.19	0.39	0.68
Day 84	4.33	4.84	3.81	0.18	0.06	0.003
Day 85	4.35	3.92	3.78	0.19	0.06	0.56
All periods	4.63	4.06	3.34	0.14	0.002	0.30

^a^ PHA = phytohemagglutinin (mm). All values are measured in mean optical density. Based on orthogonal contrast for equally spaced treatments.

**Table 8:** Effects of added dietary molybdenum on carcass characteristics of goat kids.

Item		Mo, ppm		SEM	Linear ^a^	Quad
	0	5	10			
Liver, kg	36.6	37.0	37.3	1.25	0.69	0.97
HCW, kg	17.0	17.0	17.5	0.71	0.61	0.77
CCW, kg	16.5	16.4	16.9	0.66	0.69	0.68
Shrink, %	2.7	3.5	3.4	0.34	0.16	0.29
DP, %	46.4	45.8	46.9	1.06	0.72	0.54
ADFT, cm	0.17	0.12	0.23	0.03	0.21	0.61
BF, cm	0.17	0.17	0.15	0.09	0.87	0.06
BWF, cm	0.51	0.74	0.53	0.09	0.86	0.06
LMA, cm^2^	18.0	16.3	16.8	0.75	0.29	0.23
Selection criteria	2.14	1.89	2.05	0.33	0.85	0.62

^a^ Based on orthogonal contrast for equally spaced treatments. HCW = hot carcass weight; CCW = chilled carcass weight; ADFT = adjusted fat thickness; BF = (back fat) fat depth over longissimus muscle between the 12th and 13th ribs; BWF = adjusted body wall fat; LMA = longissimus muscle area.

## Data Availability

The data presented in this study are available from the corresponding author upon request.
